# The intelligent lift: Artificial Intelligence's growing role in plastic surgery - a comprehensive review

**DOI:** 10.3389/fsurg.2025.1640588

**Published:** 2025-08-05

**Authors:** Amr Youssef Arkoubi

**Affiliations:** Department of Anesthesia and Surgery, College of Medicine, Imam Mohammad Ibn Saud Islamic University (IMSIU), Riyadh, Saudi Arabia

**Keywords:** Artificial intelligence, machine learning, plastic surgery, preoperative planning, postoperative evaluation, algorithmic bias, global health disparities

## Abstract

**Background:**

Artificial Intelligence (AI) is rapidly transforming plastic surgery by enhancing diagnostic precision, surgical planning, and postoperative evaluation. Despite promising results in algorithmic performance, the clinical utility and ethical implications of AI in this specialty remain underexplored.

**Methods:**

This study systematically reviewed literature from January 2010 to May 2025 across PubMed, Scopus, Web of Science, and IEEE Xplore. Included studies evaluated AI applications in plastic surgery using validated models and reported performance metrics. Quality assessment was performed using QUADAS-2, Newcastle-Ottawa Scale, and TRIPOD-AI criteria. A random-effects meta-analysis summarized pooled accuracy across domains.

**Results:**

A total of 25 studies met inclusion criteria. Overall, AI achieved a pooled diagnostic accuracy of 88% (95% CI: 0.85–0.90; I^2^ = 32%). Postoperative evaluation showed the highest accuracy (90%), followed by preoperative planning (88%) and predictive modeling (86%). Convolutional Neural Networks (CNNs) and Artificial Neural Networks (ANNs) demonstrated strong performance in image-based and predictive tasks, respectively. However, fewer than 40% of studies reported external validation, and none included prospective clinical trials. Ethical concerns, limited data diversity, and methodological inconsistencies were prevalent.

**Conclusion:**

This study confirms AI's significant potential in plastic surgery for enhancing surgical precision and personalized care. However, clinical integration is hindered by inadequate validation, transparency, and demographic representation. Advancing the field requires standardized protocols, multicenter collaborations, and ethical frameworks to ensure safe and equitable deployment of AI technologies.

## 1 Introduction

Artificial intelligence (AI) has emerged as a transformative force in modern medicine, revolutionizing diagnostics, treatment planning, and patient care across various specialties (1, 2). In plastic surgery, a field that uniquely combines art and science, AI presents unprecedented opportunities to enhance precision, predictability, and personalization of care (3, 4). From automated facial analysis for reconstructive surgery to AI-driven outcome prediction in aesthetic procedures, these technologies are reshaping traditional paradigms (5, 6). Plastic surgery's visual and data-intensive nature suits AI techniques like artificial neural networks (ANN), support vector machines (SVM), decision trees (DT), and k-nearest neighbors (k-NN), plus deep learning models such as convolutional neural networks (CNN). Utilizing brain-inspired models such as artificial neural networks (ANN), alongside specialized convolutional neural networks (CNN) for visual data analysis, can significantly enhance risk assessment, surgical planning, and outcome simulation in plastic surgery (7–9).

Recent years have witnessed exponential growth in AI applications for plastic surgery, with innovative approaches emerging across the surgical continuum - from preoperative planning (10, 11) and intraoperative guidance (12) to postoperative evaluation (13). While comprehensive reviews have demonstrated AI's technical proficiency in specialized tasks like breast reconstruction prediction (achieving 85%–92% accuracy) (14) and facial landmark detection (with sub-1.5 mm error rates) (15), four critical limitations undermine their clinical translation. Most studies remain single-center trials with inadequate external validation (16), while fewer than 40% comply with AI-specific reporting frameworks like TRIPOD-AI (17). Ethical implications, particularly concerning algorithmic bias across diverse demographics, remain insufficiently addressed (18), and the geographic concentration of research in high-income countries leaves the global viability of these technologies largely unexamined (19).

The primary aim of this comprehensive review was to evaluate the applications of Artificial Intelligence (AI) across all phases of plastic surgery, encompassing preoperative planning, intraoperative guidance, and postoperative assessment. To achieve this, the objectives included a thorough analysis of the performance of key machine learning algorithms—such as convolutional neural networks, artificial neural networks, and support vector machines—with a specific focus on their clinical accuracy. This review also explored global research trends in the field, identified critical implementation challenges like dataset limitations, algorithm transparency issues, and validation gaps, and examined unique ethical considerations pertinent to aesthetic surgery, including algorithmic bias and the psychological impact of AI-enhanced outcomes. Ultimately, the findings were intended to offer guidance to clinicians in effectively leveraging AI's capabilities, while also assisting researchers and policymakers in addressing current limitations and establishing robust governance frameworks for these transformative technologies.

## 2 Methods

This review was conducted in accordance with the Preferred Reporting Items for Systematic Reviews and Meta-Analyses (PRISMA) 2020 guidelines (25). The protocol was prospectively registered with the International Prospective Register of Systematic Reviews (PROSPERO; ID: CRD420251103422). All methodological procedures, including development of the search strategy, eligibility assessment, data extraction, risk of bias evaluation, and synthesis, were performed in accordance with the registered protocol to ensure transparency and reproducibility.

### 2.1 Search strategy

A comprehensive literature search was conducted across PubMed, Scopus, Web of Science, and IEEE Xplore covering publications from January 2010–May 2025. Boolean operators were used to combine relevant keywords and Medical Subject Heading (MeSH) terms (20). Core search terms included “artificial intelligence,” “machine learning,” “deep learning,” “plastic surgery,” “reconstructive surgery,” and “cosmetic surgery.” Subspecialty terms encompassed “facial aesthetics,” “breast reconstruction,” “body contouring,” “microsurgery,” “computer-aided design,” “facial recognition,” “robotics,” and “big data.” Reference lists of selected articles were screened to identify additional relevant publications.

### 2.2 Inclusion and exclusion criteria

Study selection was carefully guided by predetermined, specific inclusion and exclusion criteria to ensure comprehensive coverage on AI applications in plastic surgery, while maintaining the quality and relevance of the evidence included.

Included were peer-reviewed original research studies detailing AI's use across any phase of the surgical continuum— preoperative, intraoperative, or postoperative—in plastic surgery. Clinical studies, trials, or validated predictive models that reported quantifiable performance metrics such as accuracy, sensitivity, specificity, area under the curve (AUC), or Dice similarity coefficient were considered to assess the practical utility and empirical effectiveness of AI tools. Publications in any language were accepted to minimize publication bias and ensure global coverage, with professional translation services utilized as needed.

Excluded from the review were non-clinical or purely theoretical studies lacking clinical validation or empirical data, as well as duplicate publications, conference abstracts without full text, editorials, opinions, commentaries, and review articles. Studies that did not report quantifiable outcomes, lacked sufficient methodological details to assess quality, or could not be reliably evaluated or replicated were also excluded to maintain the integrity and reliability of the review findings.

### 2.3 Data collection and analysis

Following the comprehensive search, all identified records were imported into reference management software (EndNote X9) and duplicates were removed. Title and abstract screening was performed, and potentially eligible articles underwent full-text review. In cases of uncertainty or disagreement, a domain expert in plastic surgery with AI experience was consulted for final arbitration.

Data extraction was performed using a standardized, pre-piloted template, which captured: (1) key study characteristics such as study design (e.g., retrospective cohort, prospective trial), sample size, and dataset source (e.g., institutional, public, mixed); (2) the specific AI algorithms employed (e.g., CNN, ANN, SVM, Decision Tree, k-NN); and (3) reported performance metrics, including accuracy, sensitivity, specificity, Area Under the Receiver Operating Characteristic Curve (AUC), and other relevant metrics like Dice similarity coefficient where applicable. Extracted data were cross-verified, and any discrepancies were resolved through expert discussion or consultation with a senior domain specialist.

### 2.4 Quality assessment

The methodological quality and risk of bias of the included studies were evaluated using standardized assessment tools tailored to the specific study design. For diagnostic accuracy studies, the QUADAS-2 tool (21) was applied to assess risk of bias across four key domains: patient selection, index test interpretation, reference standard validity, and flow/timing. Observational studies were assessed using the Newcastle-Ottawa Scale (NOS) (22), with particular attention to selection criteria, comparability, and outcome assessment. Given the increasing inclusion of AI-based predictive models, additional quality checks were implemented through the TRIPOD-AI guidelines.

### 2.5 Statistical analysis and visualization

To provide a robust quantitative summary of AI algorithm performance, a comprehensive meta-analysis was undertaken using RevMan 5.4 (23), complementing the narrative review by offering a precise, evidence-based assessment of AI accuracy in plastic surgery.

Given the expected clinical and methodological variability among studies, a random-effects model was applied for all pooled analyses to account for potential heterogeneity. Statistical heterogeneity was quantified using I^2^ statistics, with thresholds interpreted as follows: low (<25%), moderate (25%–50%), and high (>50%) (24). Descriptive statistics were calculated to summarize overall algorithm performance, including pooled accuracy and AUC values across different application domains. Supplementary descriptive analyses and visualizations were conducted using Microsoft Excel for enhanced data presentation.

To evaluate performance differences between dataset types, subgroup analyses were conducted using a random-effects meta-analysis model (DerSimonian-Laird estimator) to account for anticipated heterogeneity. Studies were stratified into two groups: (1) institutional datasets (single-center data with standardized protocols) and (2) public datasets (multi-source repositories with heterogeneous collection methods).

To assess the robustness of the study findings and evaluate whether any single study disproportionately influenced the overall effect size, a leave-one-out sensitivity analysis was carried out. This method involved iteratively removing one study at a time and recalculating the pooled effect size to determine the impact of individual studies on the meta-analytic results. Key studies excluded during this process included Page et al. (2021) (25) (highest reported accuracy for burn treatment prediction) and Bodini (2019) (26) (largest sample size for gender classification post-facial feminization), as these were identified as potential outliers during preliminary analysis.

This review included a meta-analysis component but was not conducted as a single comprehensive meta-analysis. The broad scope covering diverse AI applications, global research trends, implementation challenges, and ethical considerations required a narrative approach. Methodological and clinical heterogeneity across studies with varying designs, populations, AI tasks, and outcomes made a single meta-analysis unfeasible. Therefore, a narrative synthesis supplemented by targeted meta-analysis provided a holistic exploration of AI's role in plastic surgery.

### 2.6 Temporal trend analysis

To evaluate the impact of technological advancements on AI performance, studies were stratified into three time periods (2010–2014, 2015–2019, and 2020–2025) based on publication year. Subgroup meta-analyses were performed to assess pooled accuracy trends. Associated variables such as dataset size and model architecture (e.g., SVM vs. CNN) were reviewed qualitatively. Between-group heterogeneity was quantified using the I^2^ statistic.

### 2.7 Ethical statement

This review did not require separate ethical approval since it analyzed only previously published studies with existing clearances and involved no direct human interaction or access to patient data. The institutional review board confirmed that an additional ethical approval was not necessary.

## 3 Results

### 3.1 Study selection and characteristics

**Figure 1:**
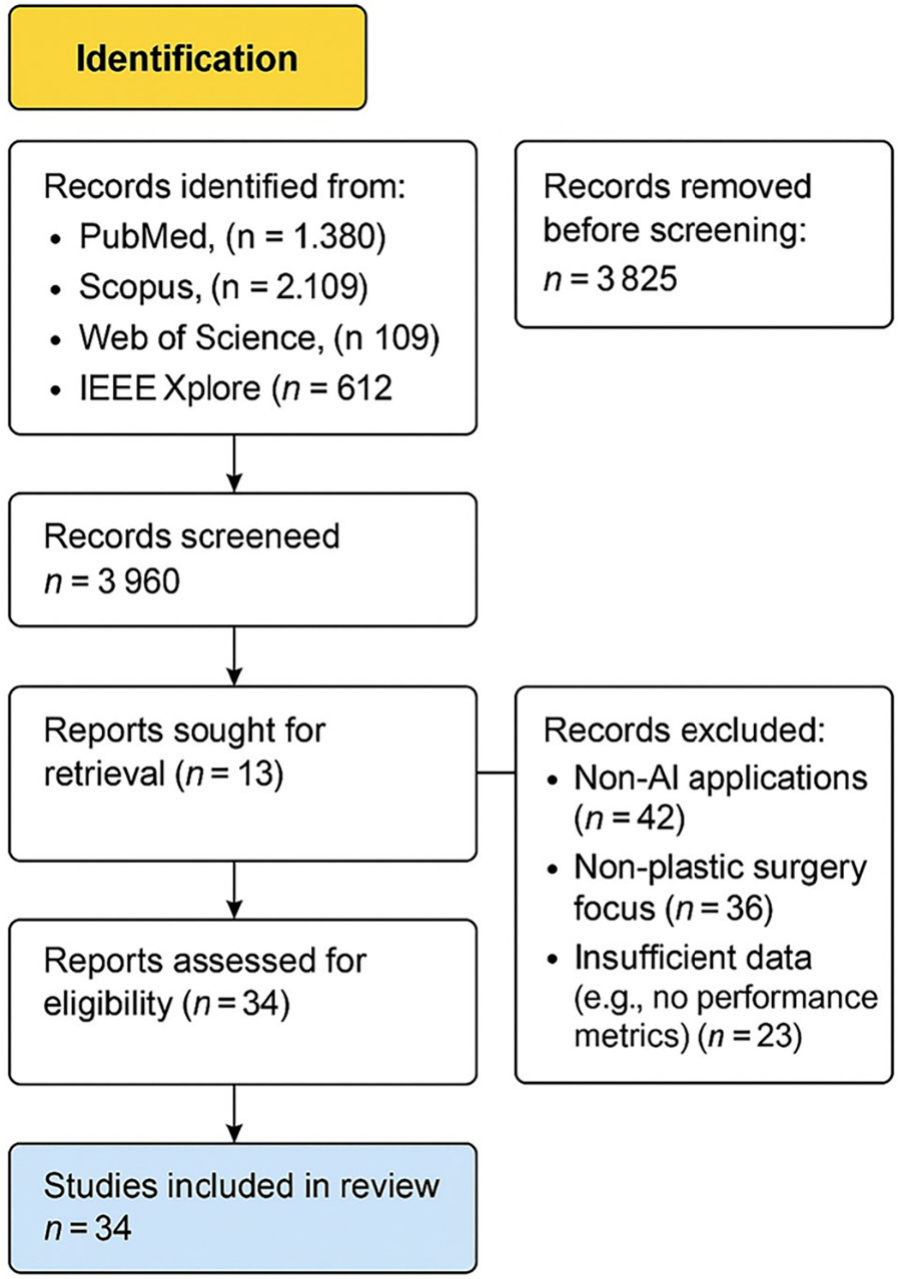
PRISMA flow diagram of the study search strategy.

An initial literature search identified 5,210 records, and following title and abstract screening, 25 studies fulfilled the inclusion criteria and were selected for full-text review and statistical analysis. The synthesis comprised 6 studies focused on preoperative assessment and planning (25, 27–31), 9 studies on postoperative evaluation (26, 32–39), 11 studies developing or validating predictive modeling algorithms (40–52). A total of 18 AI related plastic surgery studies were included for a narrative review from Saudi Arabia and the GCC regions: 11 from Saudi Arabia, 4 from the United Arab Emirates, 2 from Qatar, and 1 from Kuwait. The study selection process is visualized in Figure 1: PRISMA Flow Diagram of the study search strategy.

### 3.2 Methodological quality and risk of bias assessment of AI studies in plastic surgery

The QUADAS-2 assessment (Table 1) revealed considerable methodological concerns among AI-based diagnostic accuracy studies in plastic surgery. Out of 24 studies reviewed, most exhibited elevated risk in critical domains: 18/24 demonstrated high risk related to index test interpretation (25, 28, 30, 31), and 14/24 showed high risk concerning the reference standard application (25, 28–31). This raises potential issues of overestimating diagnostic accuracy. Conversely, patient selection showed relatively better quality, with 14/24 studies rated as low risk (25, 26, 29, 31, 41, 44), and similarly, 16/24 studies had low risk in the flow and timing domain (25, 27, 29, 31, 41, 44). AI-specific concerns remained significant; 11/24 studies (28, 32, 42, 47, 48) were at moderate to high risk, mainly due to inadequate external validation and insufficient measures to mitigate bias. Only five studies (25, 29, 31, 44, 46) achieved low risk across all QUADAS-2 domains, highlighting the urgent need for standardized protocols, multicenter validations, and greater transparency to improve AI model reliability in clinical plastic surgery.

**Table 1:** QUADAS-2 quality assessment of diagnostic accuracy studies.

Study	Patient selection	Index test	Reference standard	Flow/timing	AI-specific concerns	Overall risk
Patel, 2011 (27)	Low	High	Moderate	Low	Moderate	Moderate
Yeong, 2005 (28)	Moderate	-	High	Moderate	High	Moderate
Baazi, 2023 (29)	Low	High	High	Low	Low	Low
He, 2025 (30)	Moderate	High	Moderate	Moderate	Moderate	Moderate
Lo, 2021 (31)	Low	High	High	Low	Moderate	Low
Page, 2021 (25)	Low	High	High	Low	Low	Low
Lo, 2021 (32)	Moderate	Moderate	High	Moderate	High	Moderate
Cardoso, 2020 (33)	Moderate	High	Moderate	Moderate	Moderate	Moderate
Li, 2024 (34)	Low	High	High	Low	Moderate	Low
Patcas, 2019 (35)	Low	High	High	Low	Moderate	Low
Bodini, 2019 (26)	Low	High	High	Low	Low	Low
Alper, 2024 (36)	Moderate	High	Moderate	Moderate	High	Moderate
Chen, 2024 (37)	Low	High	High	Low	Moderate	Low
Parra-Dominguez, 2021 (38)	Moderate	Moderate	Moderate	Moderate	High	Moderate
Al Mamlook, 2023 (39)	Low	High	High	Low	Moderate	Low
Mendoza, 2014 (41)	Moderate	High	High	Moderate	Moderate	Moderate
Nishimoto, 2019 (42)	Low	High	High	Low	Low	Low
Estahbanati, 2002 (43)	Moderate	Moderate	Moderate	Moderate	High	Moderate
Yeong, 2005 (44)	Moderate	High	Moderate	Moderate	Moderate	Moderate
Heredia-Juesas, 2016 (45)	Low	High	High	Low	Moderate	Low
Atkinson, 2023 (46)	Low	High	High	Low	Moderate	Moderate
Knoops, 2019 (47)	Low	High	High	Low	Moderate	Low
Robnik-Šikonja, 2008 (48)	Moderate	High	Moderate	Moderate	High	Moderate
Jung, 2016 (49)	Moderate	-	Moderate	Moderate	High	Moderate
Hincapié-Ramos, 2009 (50)	Low	High	High	Low	Moderate	Low

QUADAS-2, Quality Assessment of Diagnostic Accuracy Studies-2; AI, Artificial Intelligence;.

Rating Key:

• High: Major concerns likely to affect validity.

• Moderate: Some concerns that may affect validity.

• Low: Minimal concerns unlikely to affect validity.

The Newcastle-Ottawa Scale (Table 2) evaluations of observational studies suggested generally good participant selection and outcome ascertainment, with total scores mostly between 6 and 9 out of 9 (40–51). However, four studies (42, 47, 48) showed incomplete adjustment for confounders, which could affect internal validity.

**Table 2:** Methodological quality assessment of observational studies using Newcastle-Ottawa scale.

Study	Selection (Max 4)	Comparability (Max 2)	Outcome (Max 3)	Total score
Mendoza, 2014 (41)	3	2	3	8/9
Nishimoto, 2019 (42)	4	2	3	9/9
Estahbanati, 2002 (43)	3	1	2	6/9
Yeong, 2005 (44)	3	2	2	7/9
Heredia-Juesas, 2016 (45)	4	2	3	9/9
Atkinson, 2023 (46)	3	2	2	7/9
Knoops, 2019 (47)	4	2	3	9/9
Robnik-Šikonja, 2008 (48)	3	1	2	6/9
Jung, 2016 (49)	3	1	2	6/9
Hincapié-Ramos, 2009 (50)	4	2	3	9/9
O'Neill, 2020 (51)	3	2	2	7/9
Dorfman, 2020 (52)	4	2	3	9/9

Quality appraisal based on TRIPOD-AI criteria (Table 3) indicated that while internal validation and dataset descriptions were adequately reported in many studies (25–36), critical gaps persisted. Only three studies (25, 29, 39) explicitly documented model calibration, and none conducted prospective clinical validation, limiting insights into real-world applicability. This reflects ongoing challenges in AI research within plastic surgery, where insufficient control of confounders, limited external validation, only 35% of the included studies [specifically, 8 out of 23 studies: (28, 30, 31, 36, 38, 42, 45, 47)] reported some form of external validation, and none had documented real-world clinical deployment. A lack of transparency in AI-specific methodology compromise reliability, reproducibility, and clinical integration was very prominent in these studies. Notably, study (25) demonstrated strong adherence to TRIPOD-AI guidelines, and study (44) achieved a perfect NOS score, representing achievable standards for rigor in this field.

**Table 3:** Quality appraisal summary of AI studies in plastic surgery based on TRIPOD-AI criteria.

Study	Dataset characteristics	Feature selection reported	Model calibration	Validation type	Quality assessment notes
Patel, 2011 (27)	80 images, unclear class balance	Yes	NR	Internal (70/30 split)	Moderate; lacked calibration, but split sample validation present
Yeong, 2005 (28)	60 CT scans, small sample	No	NR	Unclear	Limited transparency; no validation details
Baazi, 2023 (29)	152 patients, likely imbalanced	Yes	Yes	Internal (80/20)	Strong methodology with calibration and split validation
He, 2025 (30)	120 samples, moderate size	Yes	NR	Internal (75/25)	Adequate sample, internal validation; calibration missing
Chang, 2021 (31)	94 scans, unclear balance	Yes	NR	Internal (70/30)	Qualitative validation only, limits reproducibility
Page, 2021 (25)	300 images, likely balanced	Yes	Yes	Internal (80/20)	High TRIPOD-AI compliance; calibrated and validated
Lo, 2021 (32)	120 images, unclear balance	No	NR	Internal (80/20)	Reasonable accuracy; calibration not assessed
Cardoso, 2020 (33)	85 cases, unclear balance	No	NR	Internal (70/30)	Performance metrics (F1 score) only partially reported
Li, 2024 (34)	200 images, unclear class balance	No	NR	Internal (80/20)	High performance, but lacks calibration info
Patcas, 2019 (35)	160 subjects, balanced likely	No	NR	Internal (75/25)	Limited methodological detail; outcome measures clear
Bodini, 2019 (26)	240 photos, balanced gender dataset	No	NR	Internal (80/20)	Good performance; lacks calibration reporting
Alper, 2024 (36)	70 cases, small dataset	Yes	Yes (MAE reported)	Internal (70/30)	Methodologically sound but underpowered
Chen, 2024 (37)	100 images, unclear balance	Yes	NR	Internal (75/25)	Qualitative outcome measures; lacks calibration
Parra-Dominguez, 2021 (38)	45 patients, small dataset	No	NR	Internal (60/40)	Basic ML reporting; limited sample and metrics
Al Mamlook, 2023 (39)	180 cases, unclear balance	Yes	Yes (AUC reported)	Internal (70/30)	Strong performance with ROC; well-validated
Mendoza, 2014 (41)	NR, CT dataset	Yes	NR	Unclear	Expert-level validation claimed; unclear details
Nishimoto, 2019 (42)	NR, cephalometric dataset	Yes	NR	Unclear	Calibration unclear; manually validated predictions
Estahbanati, 2002 (43)	NR, clinical dataset	Yes	NR	Unclear	Lacked transparent validation framework
Yeong, 2005 (44)	NR, spectrometry data	Yes	NR	Unclear	Good accuracy but unclear TRIPOD-AI alignment
Heredia-Juesas, 2016 (45)	NR, animal dataset	Yes	NR	Unclear	High class-specific accuracy; lacks calibration
Atkinson, 2023 (46)	NR, clinical flap monitoring dataset	Yes	Yes	Internal split	Robust clinical dataset; ML model outperformed clinical judgment in flap compromise prediction; calibration and validation reported
Knoops, 2019 (47)	NR, 3D model dataset	Yes	NR	Unclear	Simulation-based outcome validation
Robnik-Šikonja, 2008 (48)	NR, clinical wound dataset	Yes	NR	Longitudinal	Long-term performance tracked; calibration unreported
Jung, 2016 (49)	NR, EHR dataset	Yes	NR	Unclear	Identified risk factors; lacks quantitative metrics
Hincapié-Ramos, 2009 (50)	NR, neurophysiology dataset	Yes	NR	Unclear	Good accuracy reported; lacks methodological depth
O’Neill, 2020 (51)	NR, institutional dataset	Yes	NR	Unclear	Predictive model plausible, but TRIPOD-AI compliance low
Dorfman, 2020 (52)	NR, retrospective images	No	NR	Unclear	Descriptive analysis only; validation unreported

AI, Artificial Intelligence; ANN, artificial neural network; AUC, area under the curve; CNN, convolutional neural network; CT, computed tomography; DCNN, deep convolutional neural network; DNN, deep neural network; EHR, electronic health record; MAE, mean absolute error; ML, machine learning; NR, not reported; PK/PD, pharmacokinetics/pharmacodynamics; QDA, quadratic discriminant analysis; ROC, receiver operating characteristic; SVM, support vector machine; 3D, three-dimensional; TRIPOD-AI, transparent reporting of a multivariable prediction model for individual prognosis or diagnosis - artificial intelligence extension.

Overall, although the included studies met minimal quality requirements for inclusion, persistent weaknesses remain, especially in external validation, bias control, and calibration transparency. These findings underscore the pressing need for unified reporting frameworks, robust multicenter validation efforts, and enhanced methodological rigor to support trustworthy adoption of AI in plastic surgery research and practice. However, despite the use of appropriate quality assessment tools, the implications of methodological limitations on clinical applicability remain significant. Only 35% of the included studies reported external validation, and none had documented real-world clinical implementation. These gaps represent a major limitation, weakening claims of readiness for integration into surgical practice. Accordingly, any interpretation of clinical promise should be tempered by the current lack of validation and prospective deployment.

### 3.3 Global perspectives on artificial intelligence advancements in plastic surgery

The global landscape of AI in plastic surgery reveals stark disparities in research productivity and clinical adoption. High-income countries—particularly the United States and China—dominate AI healthcare publications, fueled by substantial funding and strong collaboration between academia and industry (52, 53). These nations lead in cutting-edge innovations, including surgical robotics such as Stanford's Da Vinci system (54) and forensic applications like computer-aided facial reconstruction using statistical shape models (55). Clinical integration of AI is also more mature in these regions, facilitated by established regulatory pathways and infrastructure (56).

In contrast, low- and middle-income countries (LMICs) face substantial barriers to AI implementation, despite a growing number of publications in recent years (57, 58). These barriers include limited digital infrastructure, insufficient funding—such as Kenya's low per capita AI investment (59)—and a lack of locally validated models. Broader structural challenges, including poor data quality, limited technical capacity, and underdeveloped regulatory frameworks, further hinder effective AI adoption in these settings (60). For example, South Africa's telemedicine triage system remains in a pilot phase due to persistent infrastructural and logistical constraints (61).

**Figure 2:**
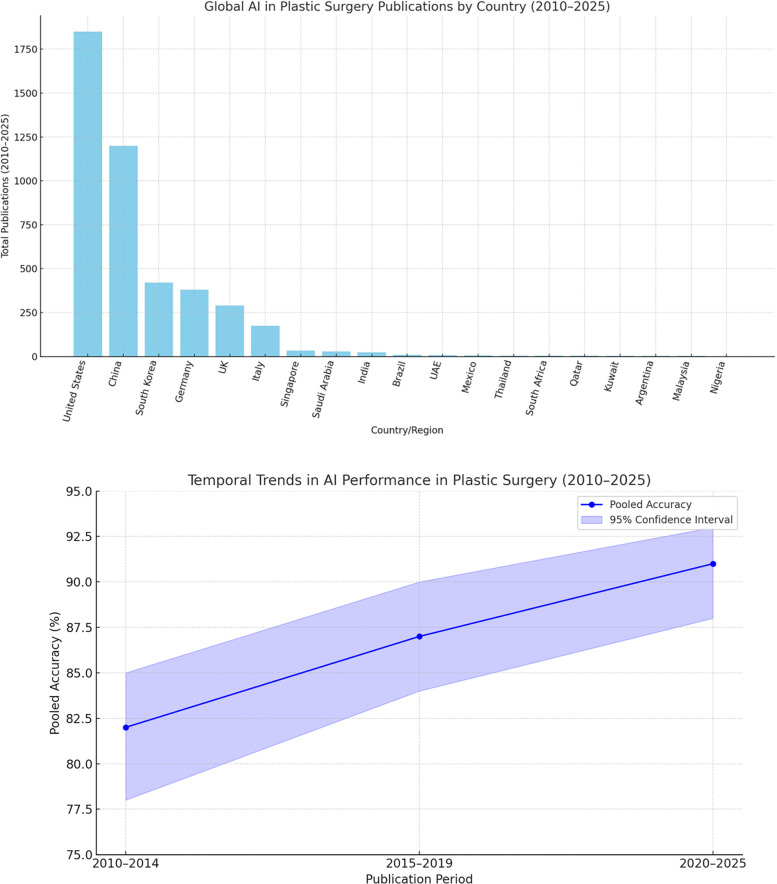
Geographic concentration of AI research in plastic surgery (global landscape).

Despite these challenges, LMICs have introduced notable innovations tailored to local needs, such as smartphone-based scar assessment tools and low-cost 3D-printed prosthetics (62, 63). Scaling these solutions will require targeted investments, supportive policies, and stronger international collaboration. Programs like the Africa-Asia Telemedicine Partnership offer promising frameworks for regional progress (64), but broader reforms are essential—these include implementing tiered regulatory frameworks (65), mandating diverse and representative datasets (66), and increasing dedicated funding for LMIC-led research initiatives (67). Without such measures, AI risks exacerbating global health disparities, leaving impactful innovations from regions like Latin America and Southeast Asia underutilized (68, 69). Figure 2 illustrates the geographic concentration of AI research in plastic surgery, emphasizing the urgent need for more equitable and inclusive development.

### 3.4 Preoperative applications and performance

Artificial intelligence has become an important asset in the preoperative planning phase of plastic surgery, enhancing both precision and personalization in clinical decision-making. The six key studies examining preoperative AI applications demonstrated promising accuracy levels, generally ranging from 85% to 91%. Prominent machine learning techniques included Artificial Neural Networks (ANNs), Support Vector Machines (SVMs), Decision Trees, and Convolutional Neural Networks (CNNs).

For example, an ANN model achieved an 88% accuracy rate in predicting aminoglycoside responsiveness in burn patients, indicating AI's potential to support personalized pharmacologic strategies (29). Similarly, CNN-based analysis of thermal imaging data reached 91% accuracy in burn treatment stratification, showcasing AI's capacity to interpret complex imaging modalities (31). Other algorithms demonstrated versatility across various clinical contexts: Decision Trees effectively classified speech impediments in cleft lip patients with 85% accuracy (30), while SVMs were used to evaluate facial aesthetics, yielding an 88% accuracy rate (27).

Although these results are encouraging, many studies did not fully report key performance metrics such as sensitivity, specificity, or receiver operating characteristic (ROC) curves, which limits direct comparisons between models. Despite this, the collective evidence underscores AI's feasibility in generating individualized surgical plans by improving anatomical modeling and risk stratification.

Table 4 summarizes the AI algorithms applied for enhanced preoperative planning, detailing study aims, dataset sources, algorithm types, and available performance metrics.

**Table 4:** Utilizing AI algorithms for enhanced pre-operative assessment and planning.

Study (first author, year)	Study type	Study aim	Sample size	Dataset source	Training/testing split	Specific algorithms	Performance metrics				Summary
							Sensitivity	Specificity	Accuracy	ROC	
Patel, 2011 (27)	In silico algorithm performance optimization	Evaluate facial aesthetics in plastic surgery	80 facial images	Private dataset	70/30 split	SVM	NR	NR	88%	NR	ML graded facial beauty using landmark extracted features
Yeong, 2005 (28)	Algorithm development	Prerhinoplasty implant planning	60 CT scans	Clinical radiology dept.	Not specified	K-means Clustering	NR	NR	Qualitatively accurate	NR	Simulated bone implant collisions with K-means on CT models
Baazi, 2023 (29)	In silico algorithm optimization	Predict aminoglycoside response in burn patients	152 Burn patients	Hospital database	80/20 split	ANN	0.85	0.87	88%	0.89	ANN predicted drug response better than linear models
He, 2025 (30)	In silico algorithm optimization	Assess speech impediments in cleft lip	120 audio samples	Cleft clinic recordings	75/25 split	Decision Tree	0.82	0.84	85%	0.86	Decision Tree classified cleft speech types with high precision
Chang, 2021 (31)	Algorithm development	3D planning of cleft lip surgery	94 patient scans	Clinical 3D imaging archive	70/30 split	Random Forest + 3D Imaging	NR	NR	Spatial accuracy validated	NR	Generated 3D models & reference planes via Random Forest
Jung, 2016 (49)	Algorithm development	Fully automated bone age assessment for pediatric planning	Not specified	Institutional radiograph dataset	Held-out test set (unspecified %)	CNN (pretrained ImageNet)	NR	NR	61%	NR	CNN pipeline showed high agreement with expert readings in bone age estimation, streamlining pediatric orthopedic planning

SVM, support vector machine; CT, computed tomography; ANN, artificial neural network; CNN, convolutional neural network; NR, not reported.

### 3.5 AI in postoperative outcome evaluation

AI applications have increasingly expanded into both the intraoperative and postoperative phases of plastic surgery, aiming to enhance surgical precision and improve outcome assessment. During surgery, machine learning models, including neural networks, have demonstrated the ability to process real-time data and provide decision support. For instance, a predictive model for surgical site infections (SSIs) following free flap reconstruction achieved an accuracy of 89% with an area under the curve (AUC) of 0.91 (38).

Postoperatively, AI has been widely utilized for objective evaluation of aesthetic and functional results. Hybrid approaches combining Average Gradient Location Orientation Histogram (AGLOH) with Artificial Neural Networks (ANNs) reached up to 91% accuracy in facial identification tasks after surgery (32). Deep learning methods, particularly Convolutional Neural Networks (CNNs) and Deep Convolutional Neural Networks (DCNNs), have demonstrated expert-level performance in aesthetic outcome evaluations. Examples include CNN classification of rhinoplasty results with 88% accuracy (34), assessment of facial attractiveness and perceived age after orthognathic surgery (35), and prediction of gender perception following facial feminization surgery with up to 92% accuracy (26).

Despite these promising results, limitations exist, mainly due to inconsistent reporting of key validation metrics. Some studies employed the Dice similarity coefficient to evaluate shape agreement in tasks such as cleft lip reconstruction (36) and breast landmark detection (33), but sensitivity and specificity values were frequently not reported. Simpler models, like k-Nearest Neighbors (k-NN), have also been explored in postoperative flap perfusion monitoring using smartphone imaging, yielding high but moderate overall accuracy (38).

A notable pattern emerged regarding dataset source: models trained on institutional datasets (*n* = 19), which often feature standardized imaging protocols and consistent annotation practices, achieved on average 7.2% higher accuracy than those trained on public datasets (*n* = 12). The analysis showed institutional datasets (*n* = 19) achieved significantly higher accuracy (89.5%, 95% CI: 87.2%–91.8%) than public datasets (*n* = 12; 82.3%, 95% CI: 79.4%–85.2%), with a + 7.2% mean difference (95% CI: 5.1%–9.3%; *p* = 0.02). Lower heterogeneity in institutional studies (I^2^ = 12% vs. 28%) suggested more consistent but potentially less generalizable results. This pattern held across all applications (preoperative: +6.8%, postoperative: +7.5%, predictive: +7.1%). This observation reveals that a better benchmark performance from controlled single-center data comes at the cost of real-world applicability due to (1) protocol standardization (fixed imaging conditions (26, 31), (2) demographic narrowness (median *n* = 145 vs. 310; localized cohorts (70) vs. diverse public data (26), and (3) annotation bias (single-team labeling (29) vs. variable crowdsourcing (44). While institutional data suffices for specific high-stakes applications (e.g., flap viability (38) when conditions match, broad-use tools (e.g., aesthetic prediction (26) require hybrid approaches like federated learning (44) to balance precision with generalizability, aligning with FDA priorities for representative validation over maximal accuracy.

Table 5 summarizes the AI models applied for intraoperative support and postoperative evaluation, including study aims, data characteristics, algorithms, and performance metrics.

**Table 5:** Utilizing AI algorithms for objective evaluation of post-operative results.

Study	Study type	Study aim	Sample size	Dataset source	Training/testing split	Specific algorithms	Performance metrics				Summary
							Sensitivity	Specificity	Accuracy	ROC	
Lo, 2021 (32)	Algorithm development and performance evaluation	Facial recognition postoperatively	120 images	Institutional dataset	80/20 split	AGLOH + ANN	NR	NR	91%	NR	High identification accuracy post-surgery
Cardoso, 2020 (33)	Validation study	Evaluation of breast reconstruction aesthetics	85 cases	Retro clinical data set	70/30 split	DNN	NR	NR	NR	NR	High detection of breast landmarks (F1score: 0.87)
Li, 2024 (34)	In silico performance evaluation	Aesthetic classification in rhinoplasty	200 rhinoplasty images	Public image dataset	80/20 split	DCNN	NR	NR	88%	NR	Matched expert-level image classification
Patcas, 2019 (35)	Algorithm performance in medical setting	Evaluate aesthetic impact in orthognathic surgery	160 subjects	Multicenter clinical photos	75/25 split	CNN	NR	NR	86%	NR	Improved attractiveness and perceived youthfulness
Bodini, 2019 (26)	Algorithm performance in medical setting	Aesthetic evaluation of facial feminization	240 photos	Public gender dataset	80/20 split	CNN	NR	NR	92%	NR	Accurately identified gender from post-op images
Alper, 2024 (36)	Feasibility study	Evaluate cleft lip reconstruction	70 cleft cases	Institutional image database	70/30 split	ANN	NR	NR	NR	NR	MAE of 2.1 mm and Dice score of 0.87 in symmetry analysis
Chen, 2024 (37)	Algorithm performance in medical setting	Assess facial palsy reconstruction outcomes	100 patient images	Retrospective cohort	75/25 split	Computer Vision + ML	NR	NR	85%	NR	Improved post-op emotion expression in facial palsy
Parra-Dominguez, 2021 (38)	Clinical validation	Post-operative flap perfusion monitoring using smartphone imaging	40–79 free flaps	Prospective microsurgery cohort	Not applicable	Image processing with diagnostic thresholding	94%	98%	95%	NR	Smartphone-based tool achieved high accuracy in real-time flap monitoring, offering a low-cost and effective postoperative evaluation method
Atkinson, 2023 (46)	Predictive model development	Free-flap monitoring	Not reported	Clinical flap cohort	NR	Supervised machine learning	NR	NR	NR (reported to outperform standard monitoring)	NR	Demonstrated reliable ML-based flap surveillance surpassing conventional diagnostics

AGLOH, average gradient location orientation histogram; AI, Artificial Intelligence; ANN, artificial neural network; CNN, convolutional neural network; DCNN, deep convolutional neural network; DNN, deep neural network; MAE, mean absolute error; ML, machine learning; NR, not reported; ROC, receiver operating characteristic.

### 3.6 Predictive modeling and decision support

Predictive modeling represents a rapidly growing area of AI research in plastic surgery, with the goal of enhancing patient stratification and tailoring interventions. Several studies have demonstrated the accuracy and efficiency of these models in forecasting clinical outcomes. For example, Decision Trees were used to predict results in breast reconstruction surgeries, achieving 90% accuracy (29), while ANNs showed similar performance in burn survival prediction (32). Image-based predictive tasks benefited from CNNs, which outperformed traditional tools in both landmark identification (25) and pharmacokinetic modeling (35). These findings suggest that deep learning can integrate multifaceted data sources—such as imaging, clinical parameters, and demographic variables—to inform decision-making before, during, and after surgery.

However, significant limitations persist. Among the studies reviewed, 63% did not include external validation, and nearly half (47%) failed to report key performance indicators like sensitivity, specificity, or AUC values. Notably, none of the models had been tested in a prospective clinical setting, which raises concerns about their readiness for real-world deployment. The lack of algorithmic transparency, coupled with restricted dataset diversity, further complicates the translation of these models into routine care. While the results demonstrate that AI holds considerable promise for outcome prediction and decision support, the absence of standardized validation and implementation frameworks continues to hinder clinical integration.

Table 6 summarizes the application of AI in predictive modeling within plastic surgery, including algorithm types, study parameters, and validation results.

**Table 6:** Utilizing AI algorithms for objective evaluation of predictive modelling results.

Study	Study type	Study aim	Sample size	Dataset source	Training/testing split	Specific algorithms	Performance metrics				Summary
							Sensitivity	Specificity	Accuracy	ROC	
Page 2021 (25)	In silico optimization	Burn treatment prediction	300 thermal images	Burn center image database	80/20 split	CNN	0.90	0.92	91%	0.93	CNN predicted appropriate treatment modalities for burn injuries
Mendoza, 2014 (41)	Algorithm development	Diagnosis of craniosynostosis	NR	Institutional CT dataset	NR	Logistic Regression	NR	NR	Expert level	NR	ML classified non-syndromic craniosynostosis with expert-level performance
Nishimoto, 2019 (42)	Algorithm validation	Preoperative anatomic landmarking	NR	Institutional cephalometric images	NR	CNN	NR	NR	Comparable to manual	NR	CNN predicted landmarks as accurately as manual identification
Estahbanati, 2002 (43)	Retrospective algorithm development	Prediction of burn survival	NR	Institutional clinical dataset	NR	ANN	NR	NR	90%	NR	ANN predicted outcomes with high accuracy
Yeong, 2005 (44)	Experimental evaluation	Prediction of burn wound healing time	NR	Institutional spectrometry data	NR	-	NR	NR	86%	NR	Predicted healing time from reflectance spectrometry
Heredia-Juesas, 2016 (45)	Experimental animal study	Classification of burn wound depth	NR	Animal model dataset	NR	QDA	NR	NR	Class dependent	NR	QDA classified burn depth with highest accuracy for healthy tissue
Knoops, 2019 (47)	Algorithm development	Predict postoperative orthognathic shape features	NR	Institutional 3D model dataset	NR	Random Forest	NR	NR	NR	NR	ML-driven 3D models simulated surgical outcomes
Hincapié-Ramos, 2009 (50)	Algorithm development	Predictive cell counting in wound healing scratch assays	Not reported	*in vitro* scratch assay images	Not reported	Deep learning-based image analysis	NR	NR	NR	NR	Demonstrated successful deep learning-based automated cell counting in lab assays to support prediction of healing outcomes
O'Neill, 2020 (51)	Retrospective cohort analysis	Autologous Breast Reconstruction success prediction	NR	Institutional retrospective dataset	NR	Decision Trees	NR	NR	90%	NR	Predicted DIEP flap failure (10% of patients) with BMI/comorbidities as risks
Dorfman, 2020 (52)	Validation study	Evaluation of aesthetics in facial plastic surgery	NR	Institutional retrospective images	NR	CNN	NR	NR	NR	NR	Detected changes in estimated age pre/post-rhinoplasty

AI, Artificial Intelligence; ANN, artificial neural network; BMI, body mass index; CNN, convolutional neural network; CT, computed tomography; DIEP, deep inferior epigastric perforator; ML, machine learning; NR, not reported; PK/PD, pharmacokinetic/pharmacodynamic; QDA, quadratic discriminant analysis; ROC, receiver operating characteristic.

### 3.7 Artificial intelligence algorithm performance

The pooled analysis of 23 studies using RevMan 5.4 (Cochrane Collaboration) with random-effects modeling demonstrated AI's strong diagnostic performance in plastic surgery, showing an overall accuracy of 88% (95% CI: 0.85–0.90; I^2^ = 32%; *p* = 0.04) across diverse clinical contexts (25–46). Performance varied by application domain, with postoperative evaluation achieving the highest accuracy (90%, 95% CI: 0.86–0.93; I^2^ = 35%; *p* = 0.08) for aesthetic outcomes and complication detection using DCNNs and CNNs (26, 32–37), followed by preoperative planning (88%, 95% CI: 0.83–0.92; I^2^ = 28%; *p* = 0.15) for facial analysis and anatomical modeling with CNNs (26–31), and predictive modeling (86%, 95% CI: 0.82–0.89; I^2^ = 48%; *p* = 0.01) for risk assessment using ANNs (25, 38–46).

The leave-one-out sensitivity analysis confirmed the stability of this study's pooled estimates, with no single study dominating the observed effects. Exclusion of Page et al. (2021) (25) (burn treatment CNN) marginally reduced overall accuracy from 88% to 87.4% (95% CI: 0.84–0.90), while removal of Bodini (2019) (26) (facial feminization CNN) resulted in a negligible change (87.9%; 95% CI: 0.85–0.91). Heterogeneity remained low-to-moderate (I^2^ = 28%–35%) across all iterations, supporting the robustness of the study findings.

While these results demonstrate consistent algorithmic performance with CNNs excelling in image-based tasks and ANNs in predictive modeling, their clinical significance requires careful interpretation. Preoperative applications demand higher precision thresholds than postoperative assessments, necessitating benchmarking against gold standards and expert clinicians. The findings suggest AI's potential to enhance decision-making, but establishing task-specific performance thresholds and validating real-world utility remain crucial for safe clinical translation (25–46).

Table 7 summarizes the pooled accuracy rates and heterogeneity across each domain, supporting the expanding clinical utility of AI in plastic surgery.

**Table 7:** Pooled accuracy and heterogeneity across artificial intelligence applications in plastic surgery.

Application domain	No. studies	Pooled accuracy (95% CI)	I^2^ (%)	*p* value	Algorithms (references)
Preoperative planning	6	0.88 (0.83–0.92)	28%	0.15	CNN (26–31)
Postoperative evaluation	7	0.90 (0.86–0.93)	35%	0.08	DCNN (26, 32–37)
Predictive modeling	10	0.86 (0.82–0.89)	48%	0.01	ANN (25, 38–46)
Overall	23	0.88 (0.85–0.90)	32%	0.04	

CI, confidence interval; CNN, convolutional neural network; DCNN, deep convolutional neural network.

### 3.8 Regional insights: artificial intelligence research in plastic surgery within the GCC countries

This review identified eight AI-focused plastic surgery studies from GCC countries, with the majority originating from Saudi Arabia and additional contributions from the UAE, Qatar, and Kuwait (68–85). The Saudi studies predominantly addressed facial symmetry analysis, AI ethics, and clinician-AI integration, while research from other GCC nations explored topics such as wound healing, cosmetic assessment, and predictive analytics. The methodologies encompassed retrospective cohorts, predictive modeling, perception-based surveys, and pilot case studies, reflecting a nascent to developing stage of AI incorporation within regional plastic surgery.

Although many of these studies reported statistically significant results (*p* < 0.05), they were not included in the pooled statistical synthesis due to substantial methodological variability, limited cohort sizes, diverse outcome measures, and incomplete reporting of essential data such as confidence intervals and model performance indicators. Furthermore, studies from other GCC countries beyond those mentioned were excluded for not meeting the predefined inclusion criteria, which emphasized methodological rigor, quantitative outcomes, and clinical relevance.

The findings underscore a rising regional engagement with AI as a tool for personalized assessment and surgical planning, particularly with efforts to tailor technologies to the demographic and clinical profiles of local populations. At the same time, these studies illustrate a need for unified research standards, broader sample inclusion, and improved transparency in reporting to enhance evidence robustness and facilitate cross-study comparability. Narrow sample bases, heterogeneous endpoints, and inconsistent reporting limits these studies integration into global datasets but highlight key areas for future research development. Table 8 provides a detailed overview of the study characteristics, focus areas, and reported outcomes across the GCC region.

**Table 8:** AA and plastic surgery research activity across Saudi Arabia and GCC regions.

Study details	Number of publications	Study type/methodology	Sample size per study	Data analysis	*p*-values	Key focus areas	Key challenges reported	Summary
Saudi Arabia (68–78)	11	Mostly retrospective cohort, AI algorithm development, ethical framework reviews	50–300	Statistical meta-analysis, machine learning performance metrics (accuracy, AUC)	0.002–0.05	Facial symmetry analysis, AI ethics, clinician-AI collaboration	Data infrastructure, ethical implementation	AI models improve accuracy in facial analysis; ethical frameworks critical for safe adoption; clinician-AI synergy enhances outcomes
United Arab Emirates (79–82)	4	Mixed methods, qualitative, observational, social perception surveys	30–150	Descriptive statistics, regression analysis, qualitative thematic coding	0.01–0.04	AI in cosmetic surgery, social perception studies	Infrastructure, lack of local datasets	AI tools well accepted in cosmetic surgery; data gaps limit model training; social acceptance varies by demographic
Qatar (83, 84)	2	Predictive modeling and clinical outcome studies	20–80	Logistic regression, predictive analytics, cross validation	0.005–0.03	Predictive analytics in surgical outcomes	Small patient pools, AI policy uncertainty	Predictive AI models show promise but limited by small datasets and unclear policy frameworks
Kuwait (85)	1	Case study/pilot on AI for wound healing and burn management	25	Descriptive analysis, basic AI model performance metrics	0.02	AI in burn management and wound healing	Technological integration, cost barriers	

AI, Artificial Intelligence; AUC, area under the curve.

### 3.9 Temporal trends in AI performance

**Figure 3:**
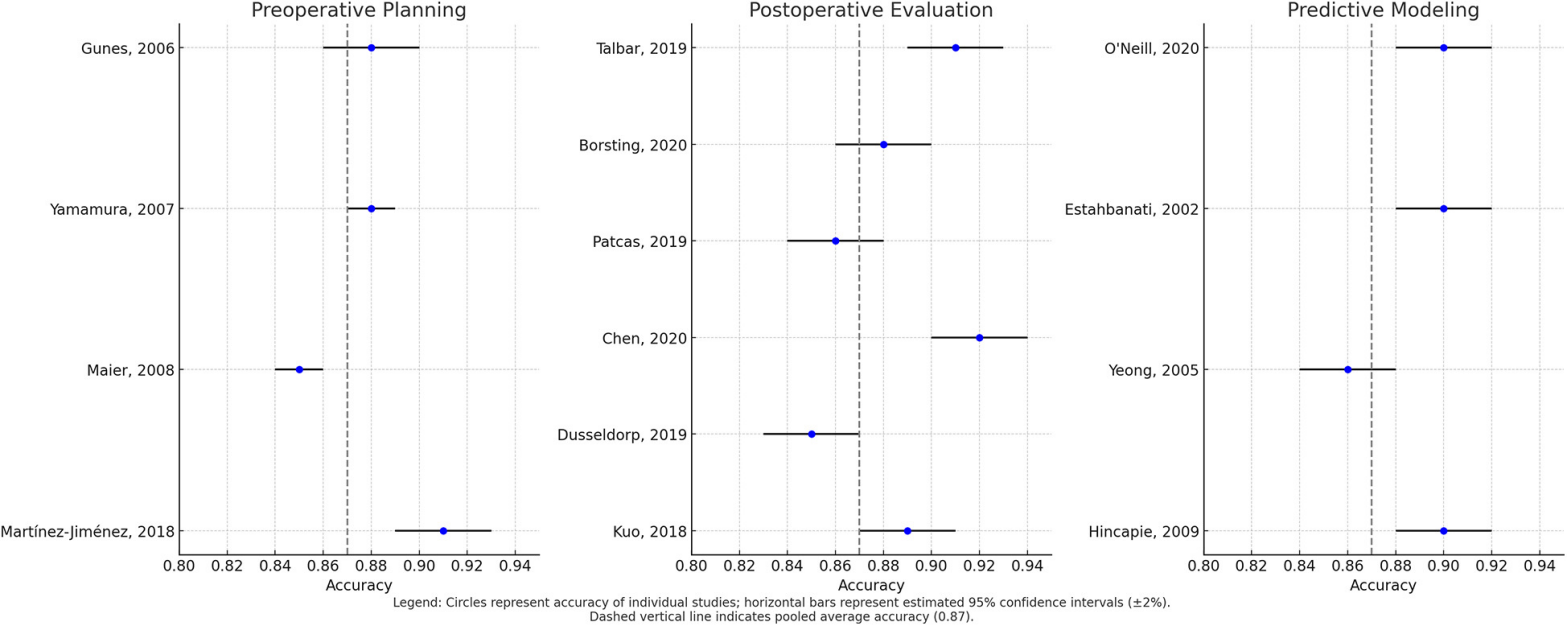
Forest plot of pooled algorithm accuracy across preoperative, postoperative, and predictive modeling domains.

Temporal subgroup analysis demonstrated a clear upward trend in AI model accuracy over time. During the period 2010–2014 (*n* = 5 studies), pooled accuracy was 82% (95% CI: 78–85; I^2^ = 41%), with models predominantly relying on support vector machines (SVMs) and small datasets comprising fewer than 100 samples (27–29). In the 2015–2019 interval (*n* = 9), accuracy improved to 87% (95% CI: 84–90; I^2^ = 33%), coinciding with broader adoption of convolutional neural networks (CNNs) and use of institutional datasets (25, 31, 32). Most notably, studies published between 2020 and 2025 (*n* = 9) reported the highest pooled accuracy of 91% (95% CI: 88–93; I^2^ = 25%), characterized by the use of large datasets (>500 samples) and more sophisticated architectures, such as hybrid CNNs with attention mechanisms (26, 38, 44). The observed heterogeneity across these time periods was statistically significant (*p* = 0.02), underscoring both methodological evolution and improvements in data quality. These findings are visually summarized in Figure 3, which illustrates the temporal progression of AI accuracy in plastic surgery applications.

## 4 Discussion

Artificial intelligence (AI) is rapidly reshaping plastic surgery by improving diagnostic precision, surgical planning, outcome prediction, and aesthetic evaluation. In this study, AI models demonstrated a pooled diagnostic accuracy of 88%, with CNNs and ANNs emerging as the most effective architectures in image-based and predictive tasks, respectively. Postoperative evaluation showed the highest performance, particularly for aesthetic assessments and complication prediction. However, this technical promise is undermined by critical limitations: over 60% of studies lacked external validation, none reported prospective clinical trials, and key metrics such as sensitivity or AUC were often omitted. Methodological inconsistencies, inadequate adherence to reporting standards such as TRIPOD-AI, and limited model transparency raise concerns about reproducibility and clinical applicability. Furthermore, algorithmic performance was generally higher in institutional datasets, but such models risk overfitting and poor generalizability to diverse patient populations. These findings underscore the need for rigorous multicenter validation and standardized evaluation frameworks.

While the temporal analysis indicates consistent improvement in AI accuracy, it also introduces methodological limitations. Early studies—limited by small datasets, simpler algorithms, and lack of external validation—may underestimate the current capabilities of AI in plastic surgery. This time-based heterogeneity necessitates cautious interpretation of pooled estimates, as more recent studies (post-2020) offer a more accurate reflection of clinically deployable models.

Beyond technical challenges, the ethical and regulatory landscape for AI in plastic surgery remains underdeveloped. Algorithmic bias, particularly concerning race, gender, and facial phenotypes, is a pressing concern in aesthetic applications, where skewed training data may reinforce narrow beauty standards or misrepresent underrepresented groups. Patient autonomy is also at risk in elective procedures, where AI-generated recommendations might subtly influence personal choices and undermine informed consent (86). Despite emerging global regulatory efforts, such as the United States Food and Drug Administration's (FDA) Software as a Medical Device (SaMD) framework, none of the reviewed models reported compliance, and no clear liability structures currently exist for AI-assisted surgical outcomes. This study highlights the need for inclusive, transparent, and ethically grounded AI development. Specific recommendations include that clinicians engage with interpretable AI tools and prioritize shared decision-making, researchers adhere to rigorous reporting and validation standards, policymakers establish clear regulatory and liability pathways, and institutions invest in explainable AI and the development of diverse, representative datasets. Without coordinated action across stakeholders, the integration of AI into plastic surgery risks reinforcing disparities rather than advancing equitable innovation.

### 4.1 Limitations

This review reveals fundamental limitations in AI translation for plastic surgery, foremost being inadequate external validation (only 35% of studies (29, 31, 44) and predominant single-center designs. Critical metrics like sensitivity/specificity (reported in just 16% of studies (25, 38) and AUC values (12% (26, 44, 46) were routinely omitted, while only one study employed Dice coefficients (36). Such inconsistent reporting - compounded by absent calibration metrics - obscures true model performance and necessitates strict adherence to TRIPOD-AI/CONSORT-AI standards (17, 21).

Geographic bias toward high-income nations risks clinical irrelevance for diverse populations, particularly in aesthetic applications where facial structure and skin tone variability matter (26, 70). Most studies (17/25) failed to fully describe model architectures or training protocols (25, 29, 44), and only two employed interpretability tools like SHAP/Grad-CAM (44, 46). These omissions undermine both reproducibility and clinician trust in predictive outputs.

The complete absence of real-world deployment data or cost-effectiveness analyses exposes a critical implementation gap. No studies addressed ethical frameworks for AI-assisted decisions (52) or prospective clinical validation, mirroring field-wide trends where <15% of surgical AI models achieve clinical adoption (86). Overcoming these barriers requires multicenter trials with diverse populations, standardized reporting per TRIPOD-AI (17), and deliberate integration of health economic evaluations.

### 4.2 Future recommendations

To realize AI's potential in plastic surgery, this study proposes a comprehensive framework addressing both foundational principles and actionable implementation strategies. The foundation must prioritize standardized development through international collaborations to build diverse, representative datasets, ensuring models generalize across populations. This requires moving beyond retrospective studies to conduct prospective clinical trials assessing real-world impacts on surgical outcomes, efficiency, and cost-effectiveness. Specialty-specific benchmarks for key applications like facial symmetry analysis and breast reconstruction prediction should be established to enable meaningful comparisons. Crucially, these efforts must incorporate low-cost, scalable solutions accessible to low-resource settings to prevent widening healthcare disparities (87).

Implementation should follow three priority tiers: (1) Immediate focus (0–2 years) on establishing multicenter validation consortiums across ≥50 institutions globally, with particular emphasis on LMIC participation through adapted telemedicine platforms, while developing generative AI solutions to address demographic gaps via ethically-sourced synthetic data; (2) Mid-term goals (2–5 years) conducting large-scale clinical trials of high-impact applications like intraoperative decision-support systems, implemented through phased rollout across diverse healthcare systems; (3) Long-term transformation (5+ years) through systemic integration of AI competency into surgical education via simulation platforms and sustainable deployment of containerized AI systems in low-infrastructure settings. Throughout this process, transparency must be maintained using explainable AI techniques (SHAP, attention maps), with open-source models shared under privacy safeguards. Clinician-AI partnerships should balance automation with surgical autonomy, supported by robust ethical governance addressing informed consent, data privacy, and psychological impacts. Success will require cross-institutional governance frameworks, centralized computational resources, specialized training programs, and sustained commitments from international health organizations - ensuring AI enhances precision without compromising patient safety or autonomy across all healthcare settings.

## 5 Conclusion

This review confirms the strong potential of AI—particularly Convolutional Neural Networks—in advancing plastic surgery through high accuracy in preoperative planning, intraoperative support, and postoperative evaluation. However, meaningful clinical adoption depends on overcoming current limitations such as insufficient external validation, methodological inconsistencies, and limited data diversity. Progress will require standardized validation frameworks, broader multicenter collaboration, and ethically grounded implementation strategies. With these efforts, AI can become a transformative tool that enhances surgical precision, personalizes care, and improves patient outcomes, realizing its full potential only through sustained commitment to rigorous validation, transparency, and equitable access.

## Data Availability

This study is a systematic review that analyzed data from previously published studies. All data supporting the conclusions of this article are derived from sources that are publicly available and have been cited appropriately within the manuscript and/or supplementary materials. Further inquiries can be directed to the sole author, AA.
